# Update 2025: Management of Non‑Small-Cell Lung Cancer

**DOI:** 10.1007/s00408-025-00801-x

**Published:** 2025-03-25

**Authors:** Hyein Jeon, Shuai Wang, Junmin Song, Harjot Gill, Haiying Cheng

**Affiliations:** 1 Department of Oncology, Albert Einstein College of Medicine, Bronx, NY 10461 USA; 2 Department of Oncology, Montefiore Medical Center, Bronx, NY 10461 USA; 3 Department of Medical Oncology, Montefiore Einstein Comprehensive Cancer Center, Bronx, NY 10461 USA; 4 Department of Medicine, Jacobi Medical Center, Albert Einstein College of Medicine, Bronx, NY 10461 USA; 5 Department of Pathology, Montefiore Albert Einstein College of Medicine, Bronx, NY 10461 USA

**Keywords:** NSCLC, Lung Cancer Management, Immunotherapy, Targeted Therapy, Actionable Genomic Alterations

## Abstract

Lung cancer remains the leading cause of cancer-related mortality worldwide. Since 2024, the non–small-cell lung cancer (NSCLC) landscape has undergone a transformative shift, driven by 11 FDA approvals. Recent advances in molecular profiling, targeted therapies, and immunotherapies have revolutionized NSCLC management, ushering in an era of personalized treatment with improved patient outcomes. The increased adoption of low-dose computed tomography (LDCT) for screening has enhanced early detection, enabling intervention at more curable stages. Molecular diagnostics now play a pivotal role in guiding treatment strategies, with actionable genomic alterations (AGAs) informing the use of *EGFR, ALK, ROS1, KRAS, NRG1,* and other targeted inhibitors in both early and advanced settings. For instance, targeted therapies are increasingly being integrated into early-stage management, with adjuvant osimertinib for *EGFR*-mutated NSCLC and alectinib for *ALK*-positive NSCLC demonstrating substantial survival benefits. Immunotherapy, particularly immune checkpoint inhibitors, has become a cornerstone of treatment for AGA-negative NSCLC, either as monotherapy or in combination with chemotherapy, and is increasingly being utilized in the perioperative setting. Furthermore, emerging therapies such as bispecific antibodies, antibody–drug conjugates (ADCs), and novel immunotherapeutic agents show promise in addressing resistance mechanisms and improving outcomes in advanced-stage disease. Although new challenges arise, the evolving NSCLC treatment paradigm continues to prioritize precision medicine, offering hope for prolonged survival and enhanced quality of life for patients.

## Introduction

Lung cancer has remained the leading cause of cancer-related mortality worldwide since the 1950s, posing a significant global health challenge [1]. NSCLC, which constitutes the majority of lung cancer cases, has historically been associated with poor outcomes due to its often late diagnosis and limited treatment options [2]. However, the landscape of NSCLC management has undergone exponential growth in recent years, driven by groundbreaking advancements in targeted therapies and immunotherapies. These innovations have ushered in a new era of precision medicine, allowing treatments to be tailored to the unique molecular and immunological profiles of patients, thereby improving efficacy and survival outcomes. Additionally, these advancements have redefined the standard of care, offering renewed hope to patients and clinicians alike. This review provides a comprehensive update on the evolving management strategies for NSCLC, with a focus on recent developments in screening, diagnostics, and therapeutic advancements in early-stage and advanced disease.

## Lung Cancer Screening

Lung cancer screening with LDCT plays a pivotal role in detecting lung cancer at earlier, more treatable stages, as evidenced by a 20% reduction in mortality demonstrated in the National Lung Screening Trial (NLST) in 2013 [3]. This resulted in a Grade B recommendation by the United States Preventive Services Task Force (USPSTF) in 2013 for annual lung cancer screening in adults aged 55–80 years with a 30-pack-year smoking history, who are either current smokers or have quit within the last 15 years [4] and a similar recommendation from the American Cancer Society (ACS) for adults aged 55–74 years old [5]. In 2020, the NELSON study in the Netherlands and Belgium published a large, randomized lung cancer screening trial which not only confirmed the relative reduction in death rate by 20% but also reinforced the importance of early detection.

These studies ultimately led to updated guidelines (Table 1) by the USPSTF in March 2021 and the ACS in November 2023, which have expanded eligibility for screening, targeting individuals aged 50–80 years with a 20 pack-year smoking history who are currently smoking or have quit within the past 15 years [6, 7]. These updates specifically aim to include underserved and lower socioeconomic status (SES) groups, as well as women and minorities who may face a heightened risk of lung cancer despite lower daily cigarette use. By reducing barriers to screening and enhancing early detection, these changes have resulted in a 61.1% relative increase in eligibility for the lowest SES quintile compared to a 49.6% increase for the highest SES quintile. However, further efforts are needed to allocate resources for tailored community outreach strategies and to address persistent barriers to screening for individuals from lower SES backgrounds. The ACS guidelines further emphasize shared decision-making and smoking cessation support to complement screening efforts, aiming to reduce lung cancer mortality and morbidity across diverse populations [7].

**Table 1:** Comparison of ACS and USPSTF lung cancer screening guideline

Criteria	ACS (2023)[7]	USPSTF (2021)[6]
Age Range	50–80 years	50–80 years
Smoking History	≥ 20 pack-years	≥ 20 pack-years
Time Since Quitting	No requirement	Within the last 15 years
Screening Method	Annual LDCT	Annual LDCT
Key Change	Removed "years since quitting" criterion	Lowered age and pack-year thresholds

While screening efforts focus on high-risk individuals with a smoking history, the increasing prevalence of lung cancer among never-smokers presents a critical area for future research and a significant public health consideration. Current risk prediction models inadequately address this population [8], necessitating the development of tools incorporating factors such as family history, second-hand smoke, occupational and environmental exposures, and genetic predispositions. Validating these models and assessing their cost-effectiveness will be essential for expanding screening efforts to never-smokers, potentially reducing lung cancer mortality in this growing demographic [8].

## Diagnostic Work-Up

### Staging

The staging of NSCLC is continually refined through evidence-based iterations led by the International Association for the Study of Lung Cancer (IASLC). Following the widespread adoption of the 8th edition since 2017 [9], which introduced more granular tumor (T) descriptors and refined stratification of metastatic (M) disease, the recently developed 9th edition has further enhanced the accuracy and clinical utility of NSCLC staging [10]. Key modifications from the 8th to the 9th edition include the reclassification of stage groups based on a more refined nodal (N) category, as well as more nuanced M descriptors that better distinguish distinct patterns of metastatic spread. These refinements reflect an expanded global database, the incorporation of more nuanced prognostic data, and the consideration of emerging treatment modalities.

Looking ahead, with the 10th edition already underway, efforts aim to build upon this trajectory by integrating emerging data sources, advanced imaging modalities, and molecular profiling tools, ultimately refining staging accuracy and better reflecting its prognostic value.

### Molecular Profiling

Molecular profiling has emerged as a cornerstone in the management of NSCLC, revolutionizing the approach to diagnosis, prognosis, and personalized treatment. Advances in molecular testing based on next-generation sequencing (NGS) have enabled the identification of key driver mutations and genetic alterations, such as *EGFR, ALK, ROS1, BRAF, MET, RET, NTRK, NRG, KRAS, and ERBB2*, that are critical for selecting targeted therapies [11]. Additionally, the discovery of biomarkers such as PD-L1 expression and tumor mutational burden has expanded the landscape of immunotherapy in NSCLC, although their role as predictive biomarkers needs further investigation [11]. Nonetheless, molecular profiling not only guides therapeutic decision-making but also provides insights into mechanisms of resistance, facilitating the development of novel treatment strategies [12]. As molecular diagnostic technologies continue to evolve, their integration into clinical practice is essential for refining prognostic accuracy, optimizing treatment outcomes, and advancing the field of precision oncology in NSCLC. Additionally, emerging evidence suggests that circulating tumor DNA analysis may aid in minimal residual disease detection, though further validation is needed.

## AGA Negative NSCLC Treatment Approaches

Immunotherapy with or without chemotherapy has been a fundamental aspect of the management of metastatic NSCLC without AGA. In recent years, immunotherapy has expanded from late-stage treatment to earlier stages of disease, leading to FDA approvals in the perioperative and adjuvant settings (Table 2).

**Table 2:** Perioperative systemic therapy for resectable NSCLC with negative actionable mutations

Drug/ Trial	Eligible NSCLC stage (AJCC edition)	Study design	Study size	Median EFS/DFS; EFS/DFS (%); HR (95% CI)	pCR:	Other outcomes	EFS based on PD-L1 score HR (95% CI)	FDA approval
*Preoperative*								
Nivolumab CheckMate 816[16]	Resectable stage IB-IIIA (7th)	Neoadjuvant nivolumab + CT vs. CT for 3 cycles	179 vs. 179	31.6 mo vs. 20.8 mo; 63.8% vs. 45.3% at 24 mo; 0.63 (0.45–0.87)	24% vs. 2.2%	OS: 82.7% vs. 70.6% at 24 mo; HR: 0.57 (0.30–1.07)	< 1%: 0.85 (0.54–1.32) 1–49%: 0.58 (0.30–1.12) ≥ 50%: 0.24 (0.10–0.61)	Mar 2022
*Perioperative*								
Pembrolizumab KEYNOTE-671[17]	Resectable stage II-IIIB (N2 stage; 8th)	Neoadj pembrolizumab + CT vs. placebo + CTx every 3 weeks for 4 cycles → surgery → adjuvant pembrolizumab vs. placebo every 3 weeks up to 13 cycles	397 vs. 400	NE vs. 17 mo; 62.4% vs. 40.6% at 24 mo; 0.58 (0.46–0.72)	18.1% vs. 4.0%	OS: 80.9% vs. 77.6% at 24 mo; HR: 0.73 (0.54–0.99)	< 1%: 0.77 (0.55–1.07) 1–49%: 0.51 (0.34–0.75) ≥ 50%: 0.42 (0.28–0.65)	Oct 2023
Durvalumab AEGEAN[18]	Resectable stage IIA-IIIB (N2 stage; 8th)	Neoadjuvant durvalumab + CTx vs. placebo + CTx every 3 weeks for 4 cycles → surgery → adjuvant durvalumab vs. placebo every 4 weeks for 12 cycles	400 vs. 402	NE vs. 25.9 mo; 63.3% vs. 52.4% at 24 mo; 0.68 (0.53–0.88)	13.0% vs. 4.3%	OS: not yet available	< 1%: 0.76 (0.49–1.17) 1–49%: 0.70 (0.46–1.05) ≥ 50%: 0.60 (0.35–1.01)	Aug 2024
Nivolumab CheckMate 77 T[19]	Resectable stage IIA-IIIB (8th)	Neoadjuvant nivolumab + CTx vs. placebo + CTx every 3 weeks for 3 cycles → surgery → adjuvant nivolumab vs. placebo every 4 weeks for 1 year	229 vs. 232	NE vs. 18.4 mo; 70.2% vs. 50.0% at 18 mo; 0.58 (0.42–0.81)	25.3% vs. 4.7%	OS: not yet available	< 1%: 0.73 (0.47–1.15) 1–49%: 0.76 (0.46–1.25) ≥ 50%: 0.26 (0.12–0.55)	Oct 2024
*Postoperative*								
Atezolizumab IMpower010[13, 14]	Resected IB-IIIA (7th)	Surgery → 1–4 cycles of CTx → atezolimumab every 3 weeks for 16 cycles or 1 year vs. best supportive care (regular scans)	507 vs. 498	42.3 mo vs. 35.3 mo; 70.2% vs. 61.6% at 24 mo; 0.79 (0.64–0.96)	NA	OS: 81.1% vs. 79.3% at 36 mo; HR: 0.995 (0.78–1.28)	< 1%: 0.97 (0.72–1.31) 1–49%: 0.87 (0.60–1.26) ≥ 50%: 0.43 (0.27–0.68)	Oct 2021
Pembrolizumab PEARLS/ KEYNOTE-091[15]	Resected stage IB-IIIA (7th)	Surgery → adjuvant pembrolizumab vs. placebo every 3 weeks for up to 18 cycles; adjuvant CTx allowed; RTx not permitted	590 vs. 587	53.6 mo vs. 42.0 mo; 67% vs. 59% at 24 mo; 0.76 (0.63–0.91)	NA	OS: 89% vs. 88% at 24 mo; HR: 0.87 (0.67–1.15)	< 1%: 0.78 (0.58–1.03) 1–49%: 0.67 (0.48–0.92) ≥ 50%: 0.82 (0.57–1.18)	Jan 2023

EFS, event-free survival; DFS, disease-free survival; pCR, pathologic complete response; HR, hazard ratio; CI, confidence interval; CT, chemotherapy; RT, radiation therapy; mo, months; OS, overall survival; NE, not estimable; mPR, major pathological response rate; *All preoperative and perioperative trials excluded patients with known EGFR or ALK alterations

### Early-Stage Resectable NSCLC

In early-stage resectable NSCLC, two pivotal studies first demonstrated the benefit of adjuvant immunotherapy. The IMpower010 trial assessed atezolizumab as adjuvant therapy after adjuvant chemotherapy in resected stage IB-IIIA NSCLC. The result showed that, in the intention-to-treat population, atezolizumab improved disease-free survival (DFS) compared to best supportive care (HR, 0.81; 95% CI, 0.67–0.99; P = 0.04). The greatest benefit was observed in the stage II-IIIA population with PD-L1 ≥ 1% (HR 0.66; 95% CI, 0.50–0.88; P = 0.0039) [13]. Notably, stage II-IIIA patients with PD-L1 < 1% did not derive a DFS benefit with atezolizumab[13]. The updated overall survival (OS) data from IMpower010 revealed an OS benefit of atezolizumab only in stage II-IIIA patients with PD-L1 ≥ 50% (HR 0.43; 95% CI, 0.24–0.78), while other subgroups did not show significant improvement [14].

The PEARLS/KEYNOTE-091 compared pembrolizumab with placebo as adjuvant therapy in resected stage IB-IIIA NSCLC. In the overall population, pembrolizumab improved DFS compared to placebo (53.6 m vs. 42.0 m; HR 0.76; 95% CI, 0.63–0.91; P = 0.0014) [15]. Unlike the IMpower010 study, the PEARLS/KEYNOTE-091 study demonstrated a DFS benefit of pembrolizumab across all PD-L1 groups. The OS data for PEARLS/KEYNOTE-091 are still pending.

Neoadjuvant immunotherapy was subsequently explored in resectable NSCLC without AGA due to several key advantages over adjuvant approaches: early control of micrometastatic disease, enhanced immune response, improved pathologic responses and reduced treatment delays. The CheckMate 816 is an open-label, randomized phase III trial that assessed neoadjuvant nivolumab plus chemotherapy versus chemotherapy alone in resectable stage IB to IIIA NSCLC. Adding nivolumab to chemotherapy prolonged event-free survival (EFS) (31.6 m vs. 20.8 m; HR 0.63; 97.38% CI, 0.43 to 0.91; P = 0.005) and resulted in a higher pathologic complete response (pCR) (24% vs. 2.2%; OR 13.94; 99% CI, 3.49 to 55.75; P < 0.001) [16].

Following the establishment of neoadjuvant chemoimmunotherapy as a standard of care for resectable NSCLC based on the results of the CheckMate 816 trial, several phase III randomized trials have further evaluated the role of immunotherapy combined with chemotherapy in the perioperative setting.

The CheckMate 77 T trial evaluated neoadjuvant nivolumab and chemotherapy compared to chemotherapy, followed by surgery and adjuvant nivolumab or chemotherapy for up to 1 year in resectable stage IIA and IIIB NSCLC. The result revealed significantly better median EFS (NR vs. 18.4 m; HR 0.58; 97.36% CI, 0.42–0.81; P < 0.001), pCR (25.3% vs. 4.7%; OR 6.64; 95% CI, 3.40–12.97) and major pathologic response (MPR) (35.4% vs.12.1%; OR 4.01; 95% CI, 2.48–6.49) in the nivolumab arm [19].

The Keynote-671 trial demonstrated that perioperative pembrolizumab plus chemotherapy in resectable stage IIA-IIIB resulted in significantly longer 24-month EFS (62.4% vs. 40.6%; HR 0.58; 95% CI, 0.46–0.72; P < 0.001), pCR (18.1% vs.4.0%; difference 14.2%; 95% CI, 10.1–18.7; P < 0.0001) and MPR (30.2% vs. 11.0%; difference 19.2%; 95% CI, 13.9–24.7; P < 0.0001) were observed. The EFS benefit favored pembrolizumab and chemotherapy across all subgroups [17].

The Neotorch trial assessed toripalimab versus placebo in combination with chemotherapy in the perioperative setting for stage II and III resectable NSCLC. The study demonstrated a benefit in the toripalimab arm with longer EFS (not estimable vs. 15.1 m; HR 0.40; 95% CI, 0.28–0.57; P < 0.001) and higher percentage of MPR (48.5% vs. 8.4%; difference 40.2%; 95% CI, 32.2–48.1; P < 0.001) as well as pCR (24.8% vs 1.0%; difference 23.7%; 95% CI, 17.6–29.8) [20].

The AEGEAN trial evaluated durvalumab vs. placebo in combination with chemotherapy in the perioperative setting for resectable stage II to IIIB NSCLC. The EFS was significantly longer in the durvalumab arm compared to placebo (HR 0.68; 95% CI, 0.53–0.88; P = 0.004). The pCR was also significantly higher, favoring durvalumab (17.2% vs. 4.3%; difference 13.0%; 95% CI, 8.7–17.6; P < 0.001) [18].

These studies collectively reinforce the efficacy of integrating immunotherapy into the perioperative setting and offer a multimodal strategy that requires early multidisciplinary discussions for resectable NSCLC. However, several important unanswered questions remain that require further investigation. For instance, determining the optimal patient selection for adjuvant therapy, including identifying prognostic factors such as pCR and MPR, is essential. In addition, there is a need to establish the correlation between surrogate endpoint and overall survival, which will require long-term follow-up for validation.

### Stage III Unresectable NSCLC

For unresectable stage III NSCLC, concurrent chemoradiotherapy remains the standard of care as a definitive treatment. The PACIFIC trial demonstrated the survival benefit of durvalumab consolidation for up to 12 months post-chemoradiation and it was approved by the FDA in 2018. The 5-year updated results were consistent with the primary analysis. Median OS was significantly longer in the durvalumab arm (47.5 m vs. 29.1 m; HR 0.72; 95% CI, 0.59–0.89), with an estimated 5-year OS rate of 42.9% for durvalumab, compared to 33.4% for placebo. Median EFS was also longer in the durvalumab arm compared to placebo (16.9 m vs. 5.6 m; HR 0.55; 95% CI, 0.45–0.68), and 5-year PFS rate was 33.1% for durvalumab compared to 19.0% for placebo. Of note, the OS benefit favored durvalumab across most subgroups, but was less clear for the PD-L1 negative cohort (HR 1.15; 95% CI, 0.75–1.75) highlighting an unmet need in this population [21, 22].

### Locally Advanced/Metastatic NSCLC

For metastatic NSCLC without AGAs, immunotherapy, either alone or with chemotherapy, has remained the standard of care for frontline treatment. Updated long-term follow-up results from several landmark trials have demonstrated significant and durable survival benefits in this population.

#### Immunotherapy: Monotherapy and Dual Combinations

In the KEYNOTE-024 and KEYNOTE-042 studies, patients with PD-L1 ≥ 50% and PD-L ≥ 1%, respectively, and without *EGFR* or *ALK* alterations were randomized to receive pembrolizumab monotherapy versus platinum-based chemotherapy as frontline therapy. In KEYNOTE-024, the median OS was 26.3 months for pembrolizumab compared to 13.4 months for chemotherapy (HR 0.62; 95% CI, 0.48–0.81). The 5-year OS was estimated to be 31.9% for pembrolizumab versus 16.3% for the chemotherapy group [23]. In KEYNOTE-042, the OS favored pembrolizumab over chemotherapy in patients with PD-L1 ≥ 50% (HR 0.68; 95% CI, 0.57–0.81), PD-L1 ≥ 20% (HR 0.75; 95% CI, 0.64–0.87), and PD-L1 ≥ 1% (HR 0.79; 95% CI, 0.70–0.89). The estimated 5-year OS was 16.6%-21.9% compared to 8.5%-10.1% across different PD-L1 groups. In the exploratory analysis, patients with PD-L1 score of 1–49% did not achieve a statistically significant OS benefit, indicating that additional treatment strategies are needed in this population[24].

A similar OS benefit was observed with cemiplimab in metastatic NSCLC with PD-L1 ≥ 50% and without *EGFR/ALK/ROS1* alterations in the frontline setting [25]. Likewise, atezolizumab demonstrated OS superiority in metastatic non-squamous and squamous NSCLC with PD-L1 ≥ 1% and *EGFR/ALK* wild-type status [26]. Long-term follow-up data are awaited to confirm the durability of these results.

The combination of dual immunotherapy with ipilimumab and nivolumab was evaluated in the CheckMate 227 trial as a frontline treatment and included patients across all PD-L1 TPS scores. The 5-year follow-up revealed a significant survival benefit. In patients with PD-L1 ≥ 1%, the 5-year OS rate was 24% for ipilimumab plus nivolumab compared to 14% for chemotherapy (HR 0.77; 95% CI, 0.66–0.91). In PD-L1 negative patients, ipilimumab plus nivolumab also demonstrated an improved 5-year OS rates (19% vs. 7%; HR 0.65; 95% CI, 0.52–0.81) [27].

#### Immunotherapy in Combination with Chemotherapy

Long-term follow-up data from key chemoimmunotherapy trials—KEYNOTE-189, IMpower150, KEYNOTE-407, and CheckMate 9LA—have further established the role of immunotherapy combined with chemotherapy as a standard of care with durable benefit in metastatic NSCLC without AGA.

In KEYNOTE-189, patients with untreated metastatic non-squamous NSCLC without *EGFR* or *ALK* alterations were randomized to receive pembrolizumab or placebo in combination with platinum and pemetrexed chemotherapy. The 5-year OS rate was 19.4 months for pembrolizumab plus chemotherapy versus 11.3 months for placebo plus chemotherapy (HR 0.60; 95% CI, 0.50–0.72). The OS benefit favored pembrolizumab plus chemotherapy across all PD-L1 groups [28].

In the IMpower150 trial, patients with metastatic non-squamous NSCLC without *EGFR* or *ALK* alterations were randomized to receive atezolizumab-carboplatin-paclitaxel (ACP), atezolizumab-bevacizumab-carboplatin-paclitaxel (ABCP), or bevacizumab-carboplatin-paclitaxel (BCP). The ABCP regimen showed an OS benefit compared to the BCP regimen (19.5mo vs. 14.7mo; HR 0.80; 95% CI, 0.67–0.95). Exploratory analysis revealed that both ABCP and ACP demonstrated significantly longer OS compared to BCP in the PD-L1-high (30.0 m vs. 26.3 m vs.15.0 m) and PD-L1-positive (22.5mo vs. 24.4mo vs. 16.0mo) subgroups, but not the PD-L1-negative group. Notably, unlike most other trials where immunotherapy was continued for a maximum of 2 years, atezolizumab was continued until disease progression or unacceptable toxicity, suggesting a flexible approach without a definitive stopping point [29].

For metastatic squamous NSCLC, the KEYNOTE-407 trial randomized patients to receive pembrolizumab or placebo in combination with carboplatin and paclitaxel or nab-paclitaxel for four cycles, followed by pembrolizumab or placebo. The estimated 5-year OS rates were 18.4% vs 9.7% (HR 0.71; 95% CI, 0.0.59–0.85), and the OS benefit favored pembrolizumab plus chemotherapy across all PD-L1 subgroups [30].

In the CheckMate 9LA trial, metastatic NSCLC patients without sensitizing *EGFR* or *ALK* alterations were randomized to receive ipilimumab and nivolumab in combination with chemotherapy or chemotherapy alone. The 5-year OS favored the ipilimumab plus nivolumab plus chemotherapy regardless of PD-L1 status (5-year OS rates: 18% vs. 11%; HR 0.73; 95% CI, 062–0.85) [31].

## AGA Directed Therapy in NSCLC

Targeted therapies have revolutionized the management of NSCLC, most notably in the metastatic setting, and are now increasingly being studied for incorporation into earlier stages, including the neoadjuvant and adjuvant settings [32]. We will explore how AGA-directed therapies are integrated into clinical decision-making across the spectrum of NSCLC and then examine the key genetic alterations guiding precision treatment in the metastatic setting.

### Early-Stage Resectable NSCLC with AGA

To date, the FDA has approved two targeted agents as adjuvant therapy —osimertinib for *EGFR*-mutated and alectinib for *ALK*-mutated tumors—for resectable, early-stage NSCLC with AGA. In the ADAURA trial, patients with stage IB–IIIA (AJCC 7th edition) NSCLC harboring *EGFR exon 19* deletion or *exon 21 L858R* mutations who had undergone surgical resection were randomized to receive three years of osimertinib versus placebo. The osimertinib arm demonstrated a marked improvement in DFS, reaching 65.8 months compared to 28.1 months in the placebo group (HR 0.27; 95% CI 0.21–0.34), and a 5-year OS of 88% versus 78% (HR 0.49; 95% CI, 0.34–0.70) [33]. Similarly, the ALINA trial evaluated patients with resected stage IB–IIIA (AJCC 7th edition) *ALK*-positive NSCLC randomized to receive either two years of alectinib or four cycles of platinum-based chemotherapy. At three years, DFS in the alectinib arm was 88.7%, compared to 54.0% in the platinum-based chemotherapy group (HR 0.24; 95% CI, 0.13–0.43) [34]. Although immunotherapy has improved outcomes in many subsets of NSCLC, it is generally considered less effective in tumors harboring driver genomic alterations such as *EGFR* and *ALK*, further emphasizing the importance of molecularly targeted therapies in these settings.

### Stage III Unresectable NSCLC with AGA

The LAURA study investigated the use of osimertinib until disease progression versus placebo in patients with unresectable stage III NSCLC harboring *EGFR exon 19* deletion or *exon 21 L858R* mutations [35]. Notably, PFS was significantly improved with osimertinib (39.1 m vs. 5.6 m; HR 0.16; 95% CI, 0.10–0.24), and at three years, OS was 84% versus 74% (HR 0.81; 95% CI, 0.42–1.56), findings that led to FDA approval of osimertinib in the adjuvant setting after chemoradiation [35]. Although the indefinite treatment duration may be unsettling, these results highlight substantial patient benefit given the poor PFS in the placebo arm and the high incidence of distant metastases, including CNS involvement. Nevertheless, further efforts are needed in this adjuvant space to achieve a truly curative outcome.

### Locally Advanced/Metastatic NSCLC with AGA

As molecular profiling becomes increasingly nuanced and comprehensive, a growing number of phase III trials have demonstrated the efficacy of targeted therapies for locally advanced and metastatic NSCLC, both as monotherapy and in combination with chemotherapy or other targeted agents (Table 3). These advancements extend beyond traditional tyrosine kinase inhibitors (TKI) to include emerging treatment modalities such as bispecific antibodies and ADCs further expanding the therapeutic arsenal for patients with AGA.

**Table 3:** Key trials supporting FDA-approved targeted therapies for advanced-stage NSCLC with actionable genomic alterations

Drug/ Major Trial (phase)	Study population	Study design	Study size	Primary endpoints	Median PFS (mo); PFS (%); HR (95% CI)	Median OS; OS (%); HR (95% CI)	Other outcomes	FDA approval month
*EGFR mutation*								
Amivantamab, Lazertinib MARIPOSA (III)[36]	Advanced untreated with *EGFR Ex19del/L858R*	Av + La vs Osi vs. La	429 vs. 429 vs. 216	PFS (Av + La vs. Os)	23.7 vs. 16.6 mo; 48 vs. 34% at 24mo; 0.70 (0.58–0.85)	NE vs. NE; 74 vs. 69% at 24mo; 0.80 (0.61–1.05)	ORR: 86 vs. 85%	Aug 2024
Amivantamab, Lazertinib MARIPOSA-2 (III)[37]	Advanced pretreated with Osimertinib with* EGFR Ex19del/L858R*	Av + La + CT vs. Av + CT vs. CT	263 vs. 263 vs. 131	PFS	8.3 vs. 6.3 vs. 4.2 mo; 37 vs. 22 vs.13% at 12 mo; Av + La + CT vs. CT: 0.48 (0.36–0.64), Av + CT vs. CT: 0.44 (0.35–0.56)	Median OS and % NR; Av + La + CT vs. CT: 0.77 (0.49–1.21), Av + CT vs. CT: 0.96 (0.67–1.35)	ORR: 63 vs. 64 vs. 36%	Sep 2024
Amivantamab PAPILLON (III)[38]	Advanced untreated with *EGFR Ex20ins*	Amivantamab + CT vs. CT only	153 vs. 155	PFS	11.4 mo vs. 6.7 mo; 31 vs. 3% at 18 mo; 0.40 (0.30–0.53)	NE vs. 24.4%; 72 vs. 54% at 24 mo; 0.67 (0.42–1.09)	ORR: 73 vs. 47%	Mar 2024
Osimertinib FLAURA-2 (III)[39]	Advanced untreated *EGFR Ex19del/L858R*	Osi + CT vs. Osi	279 vs. 278	PFS	25.5 vs. 16.7 mo; 57 vs. 41% at 24 mo; 0.62 (0.49–0.79)	NE vs. 36.7 mo; 80 vs. 72% at 24 mo; 0.75 (0.57–0.97)	ORR: 83 vs. 76%	Feb 2024
*ALK fusion*								
Alectinib ALEX (III)[40]	Advanced untreated with *ALK*	Alectinib vs. crizotinib	152 vs. 151	PFS	34.8 vs. 10.9 mo; % NR; 0.43 (0.32–0.58)	NE vs. 57.4 mo; 62.5 vs. 45.5% at 60 mo; 0.67 (0.46–0.98)	ORR: 82.9 vs. 75.5%	Nov 2017
Brigatinib ALTA-1L (III)[41]	Advanced untreated with *ALK*	Brigatinib vs. crizotinib	137 vs. 138	PFS	24.0 vs. 11.1 mo; 43% vs. 19% at 36 mo; 0.48 (0.35–0.66)	NE vs. NE; 66 vs. 60% at 48 mo; 0.81 (0.53–1.22)	ORR: 71 vs. 60%	May 2020
Ensartinib eXALT3 (III)[42]	Advanced untreated with *ALK*	Ensartinib vs. crizotinib	143 vs. 147	PFS	25.8 vs. 12.7 mo; 0.51 (0.35–0.72)	NE vs. NE; 78 vs. 78% at 24 mo; 0.91 (0.54–1.54)	ORR: 74 vs. 67%	Dec 2024
Lorlatinib CROWN (III)[43]	Advanced untreated with *ALK*	Lorlatinib vs. crizotinib	149 vs. 147	PFS	NE vs. 9.1 mo; 60 vs. 8% at 60 mo; 0.19 (0.13–0.27)	NE vs. NE; % NR; 0.72 (0.41–1.25)	ORR: 76 vs. 58%	Mar 2021
*ROS1 fusion*								
Crizotinib PROFILE 1001 (I)[44]	Locally advanced or metastatic ROS1 rearrangement-positive NSCLC	Study arm	53	ORR	19.3 mo; % NR	51.4 mo; 51% at 48 mo	ORR: 72%	Mar 2016
Entrectinib ALKA-372–001 (I), STARTRK-1 (I), STARTRK-2 (II)[45]	Advanced or metastatic ROS1 fusion-positive NSCLC receiving at least 600 mg of entrectinib every day for at least 12 mo follow-up	Study arm	53	ORR	19.0 mo; % NR	NE; 82% at 18 mo	ORR: 77%	Aug 2019
Repotrectinib TRIDENT-1 (I/II)[46]	Advanced untreated and treated with *ROS1*	ROS1-TKI untreated vs. treated vs. no CT	71 vs. 56	ORR	35.7 vs. 9.0 mo; 77 vs. 41% at 12 mo	NE vs. 25.1 mo; 91 vs. 69% at 12 mo	ORR: 79 vs. 38%	Nov 2023
*BRAF*^*V600E*^* mutation*								
Dabrafenib + trametinib NCT01336634 (II)[47]	Advanced untreated with BRAF^V600E^	Study arm	36	ORR	10.9 mo; 72% at 6 mo	24.6 mo; 51% at 24 mo	ORR: 64%	Jun 2017
Dabrafenib + trametinib NCT01336634 (II)[48]	Advanced treated with BRAF^V600E^	Study arm	57	ORR	9.7 mo; 65% at 6 mo	NE; 82% at 6 mo	ORR: 63.2%	Jun 2017
Encorafenib + binimetinib PHAROS (II)[49]	Advanced untreated and treated with BRAF^V600E^	Study arm untreated and treated	98	ORR	NE untreated, 9.3 mo in previously treated group; % NR	NE in both group; % NR	ORR: 75% untreated, 46% in treated group	Oct 2023
*MET mutation and amplification*								
Capmatinib GEOMETRY mono-1 (II)[50]	Advanced with *MET ex14* mutation or amplification	Study arm untreated and treated by *MET* status	364	ORR	4.2 untreated, 4.1 mo in treated group; % NR	OS NR	ORR: 40% untreated, 29% in treated group	May 2020
Tepotinib VISION (II)[51]	Advanced with *MET ex14* mutation	Study arm	99	ORR	8.5 mo; % NR	17.1 mo; % NR	ORR: 46%	Feb 2024
*RET fusion*								
Selpercatinib LIBRETTO-431 (III)[52]	Advanced untreated with *RET*	Selpercatinib vs. CT ± pembrolizumab	129 vs. 83	PFS	24.8 vs. 11.2 mo; % NR; 0.46 (0.31–0.70)	NE vs. NE; % NR; 1.04 (0.58–1.87)	ORR: 84 vs. 65%	Sep 2022
Pralsetinib ARROW (II)[53]	Advanced with *RET*	Study arm	121	ORR and safety	9.1 untreated, 17.1 mo treated group; % NR	NE in both groups; % NR	ORR: 70% untreated, 61% prior CT	Aug 2023
*NTRK fusion*								
Entrectinib ALKA-372–001 (I), STARTRK-1 (I), STARTRK-2 (II)[54]	Advanced with *NTRK* 600 mg of entrectinib	Study arm	54	ORR/DOR	11.2 mo; % NR	21 mo; % NR	ORR: 57%	Aug 2019
Larotrectinib NCT02122913 (I), SCOUT (I/II), NAVIGATE (II)[55]	Non-CNS primary Advanced with *NTRK*	Study arm	159 (12 lung cancer)	ORR	28.3 mo; 6 7% at 12 mo	44.4 mo; 88% at 12 mo	ORR: 79%	Nov 2018
*KRAS*^*G12C*^* mutation*								
Sotorasib CodeBreaK 200 (III)[56]	Advanced treated with *KRAS*^*G12C*^ without AGA	Sotorasib vs. CT	171 vs. 174	PFS	5.6 vs. 4.5 mo; 24.8 vs.10.1% at 12 mo; 0.66(0.51–0.86)	10.6 vs. 11.3 mo; % NR; 1.01 (0.77–1.33)	ORR: 28.1 vs. 13.2%	AA only in May 2021
Adagrasib KRYYSTAL-1 (I/II)[57]	Advanced treated with *KRAS*^*G12C*^	Study arm	116	ORR	6.5 mo; 29% at 12 mo	12.6 mo; 51% at 12 mo	ORR: 42.9%	Dec 2022
*HER2 (ERBB2) alteration*								
Trastuzumab deruxtecan DESTINY-Lung01[58]	Advanced treated with *HER2*	Study arm	91	ORR	8.2 mo; % NR	17.8 mo; % NR	ORR: 55%	AA Apr 2024
*NRG1 fusion*								
Zenocutuzumab-zbco eNRGy[59, 60]	Advanced treated with *NRG1*	Study arm	64 NSCLC	ORR/DOR	NR	NR	ORR: 33% DOR: 7.4mo	AA Dec 2024

Abbreviations: AA, accelerated approval; Av, amivantamab; DOR, duration of response; PFS, progression-free survival; HR, hazard ratio; CI, confidence interval; CTx, chemotherapy; La, lazertinib; Osi, osimertinib; RTx, radiation therapy; mo, months; OS, overall survival; NE, not estimable; NR, not reported; ORR, objective response rate

#### EGFR-Targeted Therapy in Advanced NSCLC

Osimertinib, an irreversible *EGFR* TKI, has emerged as the mainstay of first-line therapy for *EGFR*-mutated (*exon 19* deletion or *L858R*) advanced NSCLC. Following the full approval of osimertinib based on the AURA3 study [61] in patients who had progressed on a prior *EGFR* TKI with an *exon 20 T790M* mutation, the FLAURA study demonstrated superiority over earlier-generation *EGFR* TKIs (gefitinib and erlotinib) with an OS benefit of 38.6 months in the osimertinib group compared to 31.8 months in the comparator group (HR 0.8; 95% CI, 0.64–1) [62]. The trial also demonstrated a PFS benefit of 18.9 months in the osimertinib arm versus 10.2 months in the gefitinib or erlotinib arm (HR 0.46; 95%CI, 0.37–0.57) [63]. More recently, the FLAURA-2 study found that adding chemotherapy to osimertinib improved PFS from 16.7 to 25.5 months (HR 0.62).

Beyond osimertinib, amivantamab—an *EGFR-MET* bispecific antibody—has shown promise in *EGFR exon 20 insertion*–positive disease: the PAPILLON study reported a PFS of 11.4 vs. 6.7 months (HR 0.4; 95%CI, 0.3–0.53) for amivantamab plus chemotherapy compared to chemotherapy alone as first-line therapy [38]. Amivantamab plus lazertinib, a third generation of *EGFR* TKI, was also compared head-to-head with osimertinib in the MARIPOSA study which showed a PFS benefit of 23.7 vs. 16.6 months (HR 0.7; 95%CI, 0.58–0.85) [36]. However, a higher toxicity profile was observed in the amivantamab in combination plus lazertinib arm, with 75% vs. 43% of patients experiencing grade 3 or higher adverse events, leading to more frequent interruptions, dose adjustments, and treatment discontinuation [36]. Additionally, the MARIPOSA-2 study explored amivantamab with or without lazertinib in combination with chemotherapy after osimertinib progression, reporting PFS of 8.3, 6.3, and 4.2 months for the triple combination, double combination (amivantamab and chemotherapy), and chemotherapy alone, respectively [37]. These findings underscore the evolving treatment paradigm, with increasingly sophisticated *EGFR*-directed strategies and combination approaches improving outcomes for patients with *EGFR*-mutated advanced NSCLC.

#### ALK-Targeted Therapy in Advanced NSCLC

In *ALK*-rearranged advanced NSCLC, multiple first-line options have been approved, including crizotinib, alectinib, brigatinib, lorlatinib, and most recently, ensartinib [42]. Crizotinib’s use has diminished over time due to its limited CNS penetration, making newer-generation *ALK* inhibitors more attractive. Notably, the CROWN study demonstrated that lorlatinib delivered a median PFS that was not yet reached at five years, compared to 9.1 months in the crizotinib arm (HR 0.19; 95% CI, 0.13–0.27) [43]. Similarly, ensartinib has also recently been FDA-approved in the first-line setting, showing a PFS of 25.8 months versus 12.7 months with crizotinib (HR 0.51; 95% CI, 0.35–0.72). As the treatment landscape evolves, the diverse efficacy and side effect profiles of *ALK*-targeted agents—including the impressive results demonstrated by lorlatinib—will play a pivotal role in guiding personalized therapy decisions and optimizing outcomes for each patient.

#### Emerging Therapies for Other AGA

Repotrectinib has expanded the treatment repertoire for *ROS1*-rearranged advanced NSCLC, joining entrectinib and crizotinib as an FDA-approved option. In the TRIDENT-1 phase I/II study, repotrectinib demonstrated a remarkable PFS of 35.7 months compared to 9 months for crizotinib [46], underscoring its potential as a highly effective therapy in this molecularly defined patient population.

Encorafenib and binimetinib offer another therapeutic option for *BRAF V600E*-mutated NSCLC, demonstrating a 75% overall response rate (ORR) in untreated patients. However, treatment-related adverse events occurred in 24% of patients, leading to dose reductions or discontinuations, underscoring the need for larger studies to better understand tolerability and long-term outcomes [49].

Tepotinib was approved in February 2024, demonstrating a PFS of 8.5 months and an OS of 17.1 months, showing promise for patients with *MET* *exon 14* skipping mutations [51].

Selpercatinib received approval based on the LIBRETTO-001 study with an ORR of 84% compared to 65% in the control group [52], and pralsetinib has also gained approval for *RET*-altered NSCLC with an ORR of 70% in the treatment naïve group [53].

Entrectinib and larotrectinib remain viable options for patients harboring *NTRK* fusions, either as an initial treatment when no other alternatives exist or after prior therapies [54, 55].

Sotorasib and adagrasib have been approved based on the CodeBreaK200 [56] and KRYSTAL-1 [57] studies, respectively. However, sotorasib did not receive full approval in 2023 after its accelerated approval because, despite meeting the PFS endpoint (5.6 vs. 4.5 months), the OS was not improved (10.6 vs. 11.3 months), and concerns were raised regarding patient dropout in the docetaxel arm and early crossover to sotorasib before disease progression [56].

Trastuzumab deruxtecan-nxki (TDxd) received tumor-agnostic approval for *HER2*-positive (IHC 3 +) solid tumors. This *HER2*-directed ADCs deliver deruxtecan payload, a topoisomerase I inhibitor, directly to *HER2*-expressing tumor cells. In the DESTINY-Lung01 phase II study, TDxd demonstrated an ORR of 55%, a median DOR of 9.3 months, and a PFS of 8.2 months [58], highlighting its efficacy in this subset of patients and offering an alternative in second line treatment.

The recent approval of zenocutuzumab-zbco (zeno), based on the eNRGy study, presents an advancement for previously treated advanced NSCLC with *NRG1* fusion. Zeno is a bispecific antibody targeting *HER2*/*HER3* signaling, effectively inhibiting *NRG1* binding. While the full publication is pending, the FDA has reported an ORR of 33% and a median DOR of 7.4 months in a cohort of 64 NSCLC patients [60]. Previous abstract presentation showed 41 patients with NSCLC with an ORR 35% with good tolerability [64].

## Conclusions

**Fig. 1:**
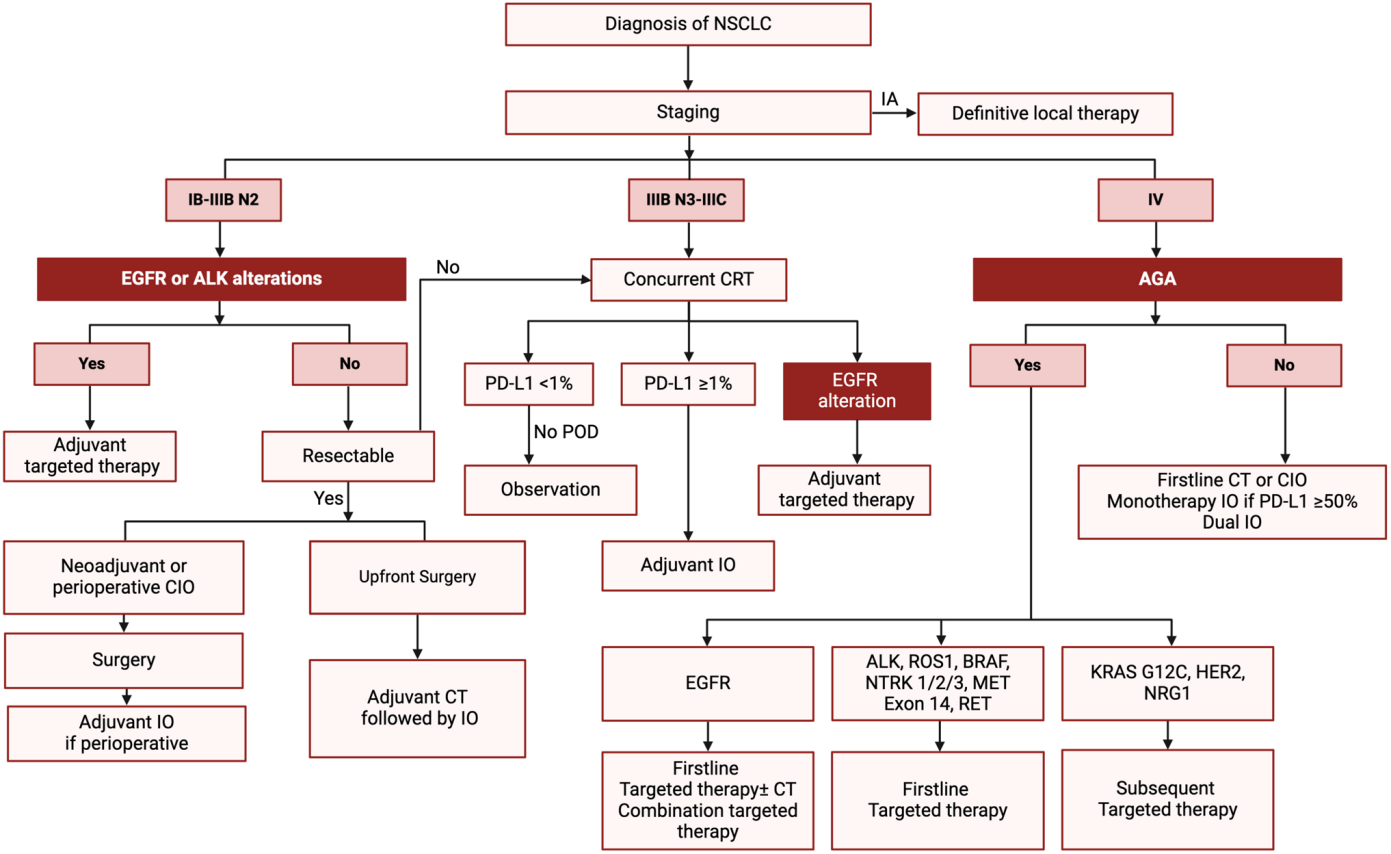
Simplified schematic of initial management of NSCLC. *AGA* actionable genomic alterations, *CIO* chemoimmunotherapy, *CT* chemotherapy, *IO* immunotherapy, *NSCLC* non-small-cell lung cancer

The management of NSCLC has evolved remarkably over the past decade, reflecting a paradigm shift driven by advances in screening, molecular diagnostics, and targeted therapeutics. Enhanced screening protocols have enabled earlier detection, while ongoing refinements in staging ensure patients receive the most appropriate treatments based on precise risk stratification. The incorporation of molecular profiling and immunotherapy into both early-stage and advanced disease settings has expanded treatment options, improved survival outcomes, and set the stage for more personalized care (Fig. 1). Targeted therapies for *EGFR, ALK, ROS1*, and other actionable alterations have revolutionized the therapeutic landscape, and novel approaches—ranging from bispecific antibodies to ADCs—continue to emerge. Although challenges remain, the future of NSCLC management is increasingly defined by innovation and collaboration.

## Data Availability

No datasets were generated or analyzed during the current study.

## Abbreviations

ABCP: Atezolizumab-bevacizumab-carboplatin-paclitaxel
ACP: Atezolizumab-carboplatin-paclitaxel
ADCs: Antibody–drug conjugates
AGA: Actionable genomic alterations
ACS: American Cancer Society
BCP: Bevacizumab-carboplatin-paclitaxel
DFS: Disease-free survival
EFS: Event-free survival
ITT: Intention to treat
IASLC: International Association for the Study of Lung Cancer
LDCT: Low-dose computed tomography
MPR: Major pathologic response
NLST: National Lung Screening Trial
NSCLC: Non-small-cell lung cancer
OS: Overall survival
ORR: Overall response rate
pCR: Pathologic complete response
SES: Socioeconomic status
TDxd: Trastuzumab deruxtecan-nxki
TKI: Tyrosine kinase inhibitors
USPSTF: United States Preventive Services Taskforce
zeno: Zenocutuzumab-zbco
